# Who Is Going for VCT? A Case Study in Urban Burkina Faso

**DOI:** 10.5402/2012/307917

**Published:** 2012-12-30

**Authors:** Fati Kirakoya-Samadoulougou, Seydou Yaro, Paulin Fao, Marie-Christine Defer, François Ilboudo, Youssouf Langani, Nicolas Meda, Annie Robert, Nicolas Nagot

**Affiliations:** ^1^Pôle Epidémiologie et Biostatistique, Institut de Recherche Expérimentale et Clinique (IREC), Faculté de Santé Publique (FSP), Université catholique de Louvain (UCL), Chapelle-aux-Champs, 30, B1. 30. 02, 1200 Bruxelles, Belgium; ^2^Centre Muraz, Bobo-Dioulasso, Burkina Faso; ^3^Institut National de la Statistique et de la Démographie, Ouagadougou, Burkina Faso; ^4^Département de Santé Publique, Université de Ouagadougou, Ouagadougou, Burkina Faso; ^5^CHRU Montpellier and Université Montpellier 1, Montpellier, France

## Abstract

*Introduction*. Voluntary HIV counselling and testing (VCT) is a key element of treatment and is essential for prevention of vertical HIV transmission. Little information is available on the uptake of VCT in Burkina Faso. This study aims to assess the prevalence of VCT in urban Burkina Faso, where the epidemic is still highly concentrated. *Methods*. We conducted a two-stage clustered population-based survey among 1,694 subjects living in Ouagadougou, Burkina Faso. After informed consent was obtained, a behavioural questionnaire was administered to participants. *Results*. Overall, 10.2% of individuals had used VCT, while 9% were women. Among women who had a child after the launch of the programme to prevent mother-to-child transmission (PMTCT), only 10.4% have been tested for HIV. Almost all participants (99.3%) were aware of HIV/AIDS, and 65% knew the main methods of prevention. In multivariate analysis, older age and being married and better educated were independent factors associated with VCT. *Conclusions*. Despite high public knowledge and awareness about HIV, VCT uptake was still very low and PMTCT coverage was poor. New strategies are required to increase VCT uptake in urban areas, in particular among the youngest age.

## 1. Introduction

Voluntary counselling and testing (VCT) plays a pivotal role within a comprehensive range of measures for HIV/AIDS prevention and support and should be strongly encouraged. VCT has become an integral part of HIV control programs in many countries. Services have evolved to reflect developments in the treatment and care for HIV-related illnesses, important in reducing HIV transmission [1]. 

The campaigns against HIV/AIDS have largely focused on community implications and healthy living. The uptake, attitudes, and perceptions of the public regarding HIV testing may provide important information for the planning of preventive and interventional programmes. 

Positive attitudes toward VCT have been demonstrated in women attending antenatal clinic (ANC) due to their concern for the health of the expected baby, self-perceived HIV risk, and knowledge of medical intervention to reduce disease symptoms or prevent vertical transmission [2]. It is not clear if these findings can be extrapolated to other population groups.

Approximately 120,000 individuals are infected with HIV in Burkina Faso, with 60% of these living in urban areas and only a small proportion of them aware of their HIV status [3]. Prevention of mother-to-child transmission (PMTCT) programmes have been launched in urban areas of Burkina in 2001.

Little information is available on the uptake of VCT and on the factors associated with the use of VCT services in the adult urban general population. 

## 2. Methods

### 2.1. Study Design

The current study was part of population-based survey conducted from January 2003 to March 2003 among 1694 women and men in general population of Ouagadougou, Burkina Faso [4]. We used a two-stage clustered sample design with the assistance of the Central Statistics Office at the Ministry of Economy and Finance in Burkina Faso. At the first stage, we randomly selected 59 administrative areas over 713 in total, and at the second stage we randomly selected 20 households in each selected area. All adults aged 15–49 years and present in the selected households were invited to participate in the study. After detailed explanations of the study and informed consent, a trained interviewer administered a questionnaire assessing sociodemographic characteristics, awareness of HIV/STI, and utilization of HIV testing services. Blood sample was also collected at home by a lab technician for anonymous HIV, HSV-2, and syphilis testing.

Female participants were invited to consult the study central laboratory for a free genital sampling to detect genital infections including trichomoniasis. 

### 2.2. Laboratory Tests

Serum samples were screened for HIV diagnosis by a first enzyme linked immunosorbant assay (ELISA) test (ABBOTT Murex, Dartford, UK), and positive results were confirmed by a second ELISA to distinguish between HIV-1 and HIV-2 infection (wellcozyme HIV Recombinant, Murex HIV-2). 

HSV-2 was detected by a type-specific HSV-2 ELISA test (KALON Biological LTD, Surrey, UK), which showed high performance in African sera. The diagnosis of active serological syphilis was based on a positive Rapid Plasma Reagin test, confirmed by the Treponema Haemagglutination Assay test (Newmarket Laboratories Ltd Germany), according to the manufacturer's instructions of both tests. We also retested all positive results (for syphilis) in a reference laboratory (Centre Muraz) with another reagents for quality control.

A vaginal swab was collected for each woman and used for inoculation in a culture media of *Trichomonas vaginalis* (InPouch TV, BioMed Diagnostics, San Jose, CA, USA). 

### 2.3. Statistical Analysis

*χ* ^2^ test, Fisher's exact test, and Student's *t*-test were used for comparisons at a significance level of 5%. Multiple logistic regression analysis was performed to identify factors associated with the use of VCT services. A cut-off *P* value of 0.15 (or factors already known to be associated with HIV infection) was used to select variables for the multivariable analysis. All the statistical analyses were performed using the Stata 9 software.

## 3. Results

### 3.1. Population Characteristics

A total of 1694 participants (among 2200 eligible people) were enrolled and interviewed between January and May 2003, with a response rate of 77%. The mean age of the study participants was 27 years (range 15 to 49), 48.6% were married, and the average number of children per women was 3.3. The majority of participants had completed primary school. Men had much better knowledge of HIV than women; they were more likely to have heard of HIV (96% versus 89%,  *P* < 0.001), to know that consistent condom use is an effective way of preventing HIV (83% versus 69%,  *P* < 0.001), to know that HIV can be sexually transmitted (94% versus 82%,  *P* < 0.001), to know that HIV can be transmitted by contact with infected blood (61% versus 51%,  *P* < 0.001), and to know that a person with HIV can feel healthy (85% versus 74%,  *P* < 0.001). 

Overall, 10.2%  (*n* = 1694)  of individuals (9.0% of females and 11.6% of males, *P* = 0.07) had used voluntary counselling and testing services (VCT) (Table 1). Only 9.6% of women who had a child after 2001 (thus who should have received antenatal HIV testing) had been tested for HIV. 

A key barrier to testing for 33.7% of responders was that they were ‘‘afraid to know” if they were HIV positive. Thirty-seven percent reported that they had no reason to believe that they were infected, and 5% did not test because they know HIV testing. There were several significant gender differences in the reported barriers to testing: women were more likely to report “don't know where can I do it” (56.6% versus 43.4%,  *P* = 0.01) and “afraid to know” (61.8% versus 38.2%,  *P* = 0.04). 

Among those who had not been tested, 80.7% reported that they wished to know their HIV status. 

### 3.2. Correlates of Having Been Tested for HIV

In univariable analysis (Table 1), using VCT services was higher among married individuals and tended to be higher among monogamous individuals versus polygamous ones (*P* = 0.10). VCT users were also older (29 ± 8 versus 26 ± 9,  *P* < 0.001) and better educated (*P* < 0.001).

 The condom use or the reported number of sexual partners did not seem to influence the uptake of VCT. 

Among HIV-infected individuals, only 12.3% ever tested for HIV. There was no significant difference in HSV-2 infection (*P* = 0.17), syphilis (*P* = 0.70), and trichomoniasis (*P* = 0.16) between these two groups (Table 1).

The multivariable analysis showed that age 20 years or older, being married, and having better education were significantly associated with VCT uptake (Table 1).

## 4. Discussion

The low prevalence (10%) of voluntary HIV testing in the general population is rather low and in accordance with other surveys conducted in 20 African countries since 2000; in the Demographic Health Surveys, a median of 10% of men and 8% of women reported ever having had an HIV test [5]. Despite its high potential benefit, there is limited understanding of factors that determine the acceptability and uptake of VCT, particularly for client-initiated testing outside health-care settings.

In Burkina Faso, few women who should have been exposed to PMTCT in Ouagadougou since 2001 were actually tested, despite a strong desire to know their HIV status. An overall prevalence of 21.6% for VCT among pregnant women was reported in 2003 by the Ministry of Health in Burkina Faso [6]. The same survey identified that a factor possibly explaining the low rate was fear of not having access to effective treatment. In South Africa individuals who had tested reported also that they would be more willing to test if antiretroviral therapy (ART) were easily available [7]. 

Antiretroviral roll-out was initiated in Burkina Faso in July of the same year this study was conducted, and therefore the influence of ART availability on VCT uptake is difficult to assess. A study among mineworkers in South Africa in 2001 found that only 14% of participants indicated that they would be more likely to use VCT services if ART were available [8]. Similarly, this study was conducted in 2001 before the roll-out of ART in South Africa, and the authors reported that ART awareness was certainly low among the study participants.

Among the reasons of nontesting, “afraid to know” could be a strong reason to decline a testing or had no reason to believe that they were infected. The evidence of high willingness to test with very limited achievement in Burkina Faso is also reported from other African countries.

Direct offer of HIV testing in a convenient location usually leads to high uptake in community settings [2]. Acceptance of provider-initiated testing can exceed 90% for antenatal clinic attendees [2] but with rates of return of only 45%–75% when a second visit is required to get the test result [2]. However, only a minority of African adults will make unsolicited visits to free-standing or clinic-based VCT centres [9]. Major disincentives include fear of breach of confidentiality, a sense of futility if testing is not linked to HIV care, and influence from a sexual partner [10]. Accessibility and cost are also important [10]. 

Classic PMTCT programmes are based primarily on systematic HIV counselling and testing among antennal clinic attendees who are then free to decline the test. The low prevalence of VCT among mothers who had a child after the implementation of PMTCT in Ouagadougou suggests that PMTCT services were not efficient. However, it is important to know that PMTCT prevention programmes in Burkina Faso only got off the ground in 2001. Widespread use of PMTCT in Ouagadougou was effective only from May 2002. 

We also found that people who had been tested for HIV were not more infected than nontested individuals, suggesting that the main motivation for testing was not driven by clinical symptoms or referral by clinicians. In the survey of Killewo et al. [11] in rural Tanzania, about 96% of those who accepted the HIV test reported the need to know their HIV status as their main motivation. Individuals who refused testing perceived themselves at low risk of infection.

HSV-2 is generally considered as a useful marker for sexual behaviours [12]. The similar prevalence of HSV-2 among VCT users and nonusers confirmed that sexual behaviour did not influence HIV testing. These findings suggest that persons at high risk of HIV do not use VCT services, which is a main concern in West Africa where the epidemic is still driven by high-risk groups. 

This study has some limitation including the reliance on respondent self-report, with potential recall bias.

In conclusion, new strategies are urgently needed to maximise HIV testing and prevention, including in the PMTCT programme in Burkina Faso. Additional efforts are also required to develop new strategies for high-risk groups. 

## Figures and Tables

**Table 1:** Sociodemographic and behavioural characteristic, and HIV/STI prevalence among subjects tested for HIV in urban Burkina Faso.

	Total *N*	Users of VCT		Univariate OR (95% CI)*	Multivariate OR (95% CI)	*P* value**
		*n*	%			
Gender						0.19
Women	899	81	9.0	1	1	
Men	795	92	11.6	1.3 (1.0–1.8)	1.1 (0.8–1.6)	
Marital status						0.003
Single	872	67	7.7	1	1	
Married or living in couple	822	106	12.9	1.8 (1.3–2.5)	2.0 (1.3–3.1)	
Polygamous						0.10
No	1540	163	10.6	1	1	
Yes	153	10	6.5	0.6 (0.3–1.1)	0.6 (0.3–1.2)	
Age (years)						
15–19	459	13	2.8	1	1	
20–29	677	89	13.1	5.2 (2.9–9.4)	4.2 (2.3–7.8)	<0.001
30–39	361	49	13.6	5.4 (2.9–10.1)	3.9 (1.9–8.2)	<0.001
40–49	197	22	11.2	4.3 (2.1–8.8)	3.2 (1.4–7.4)	0.007
Education						
None	506	26	5.1	1	1	
Primary	535	42	7.9	1.6 (0.9–2.6)	1.9 (1.1–3.2)	0.02
Post-primary	653	105	16.1	3.5 (2.3–5.5)	4.5 (2.8–7.3)	<0.001
Religion (Muslim)						
No	719	81	11.3	1	—	—
Yes	965	92	9.5	0.8 (0.6–1.1)	—	—
Never used condom with the last occasional partner						
No	343	154	44.9	1	—	—
Yes	43	19	44.2	0.9 (0.5–1.8)	—	—
No. of sexual partners (past 12 months)						0.23
1	1197	161	13.5	1	1	
2+	103	12	11.7	0.8 (0.5–1.6)	0.7 (0.3–1.3)	
HIV infection						0.65
No	1605	164	10.2	1	1	
Yes	73	9	12.3	1.2 (0.6–2.5)	0.8 (0.4–1.8)	
HSV-2 infection						0.48
No	1343	132	9.8	1	1	
Yes	330	41	12.4	1.3 (0.9–1.9)	1.2 (0.8–1.8)	
Syphilis						
No	1653	171	10.3	1	—	—
Yes	25	2	8.0	0.8 (0.2–3.2)	—	—
Trichomoniasis						
No	520	167	32.1	1	—	—
Yes	30	6	20.0	0.5 (0.2–1.3)	—	—

*Percentage (95% confidence interval).

**P-value in multivariable logistic regression.

## References

[1] World Health Organization (1999). Voluntary coun-selling and testing for HIV infection in ante-natal care: practical consideration for imple-mentation. *WHO/HIV/AIDS and Sexually Transmitted Infections Initiative*.

[2] Cartoux M, Msellati P, Meda N (1998). Attitude of pregnant women towards HIV testing in Abidjan, Cote d’Ivoire and Bobo-Dioulasso, Burkina Faso. DITRAME Study Group (ANRS 049 Clinical Trial). Diminution de la Transmission Mere Enfant du VIH. Agence Nationale de Recherches sur le SIDA. *AIDS*.

[3] UNAIDS Epidemiological fact sheets on HIV/AIDS and sexually transmitted infections. http://www.who.int/GlobalAtlas/predefinedReports/EFS2006/EFS_PDFs/EFS2006_BF.pdf.

[4] Kirakoya-Samadoulougou F, Defer MC, Yaro S (2011). Low seroprevalence of syphilis in Burkina Faso. *Sexually Transmitted Infections*.

[5] Measure DHS Measure DHS HIV/AIDS survey indicators database. http://www.measuredhs.com/hivdata/reports/start.cfm.

[6] MoH/BF Enquête sur les facteurs limitant l'adhésion des femmes enceintes au CDV. Résultats provisoires.

[7] Pettifor A, MacPhail C, Suchindran S, Delany-Moretlwe S (2010). Factors associated with HIV testing among public sector clinic attendees in Johannesburg, South Africa. *AIDS and Behavior*.

[8] Day JH, Miyamura K, Grant AD (2003). Attitudes to HIV voluntary counselling and testing among mineworkers in South Africa: will availability of antiretroviral therapy encourage testing?. *AIDS Care*.

[9] World Health Organization Report of a “lessons learnt” workshop on the six ProTEST pilot projects in Malawi, South Africa and Zambia. http://whqlibdoc.who.int/hq/2004/WHO_HTM_TB_2004.336.pdf.

[10] Nuwaha F, Kabatesi D, Muganwa M, Whalen CC (2002). Factors influencing acceptability of voluntary counselling and testing for HIV in Bushenyi District of Uganda. *East African Medical Journal*.

[11] Killewo JZJ, Kwesigabo G, Comoro C (1998). Acceptability of voluntary HIV testing with counselling in a rural village in Kagera, Tanzania. *AIDS Care*.

[12] Cowan FM, Johnson AM, Ashley R, Corey L, Mindel A (1994). Antibody to herpes simplex virus type 2 as serological marker of sexual lifestyle in populations. *British Medical Journal*.

